# Structured myeloid cells and anti-angiogenic therapy in alveolar soft part sarcoma

**DOI:** 10.1186/1479-5876-11-237

**Published:** 2013-09-27

**Authors:** Chiara Castelli, Marcella Tazzari, Tiziana Negri, Barbara Vergani, Licia Rivoltini, Silvia Stacchiotti, Silvana Pilotti

**Affiliations:** 1 Unit of Immunotherapy of Human Tumors, Fondazione IRCCS Istituto Nazionale dei Tumori, Via G. Venezian 1, Milan 20133, Italy; 2 Department of Diagnostic Pathology and Laboratory, Laboratory of Experimental Molecular Pathology, Milan 20133, Italy; 3 Consorzio M.I.A (Microscopy and Image Analysis), Università degli Studi di Milano-Bicocca, Monza 20900, Italy; 4 Department of Cancer Medicine, Adult Mesenchymal Tumor Medical Oncology Unit, Fondazione IRCCS Istituto Nazionale dei Tumori, Milan, Italy

**Keywords:** Immune infiltrating cells, Inflammation, Myeloid cells, Soft tissue sarcoma, Anti-angiogenic therapy

## Abstract

Alveolar soft part sarcoma (ASPS) is a rare soft tissue sarcoma and the clinical management of patients with unresectable, metastatic disease is still challenging. ASPS expresses an array of potentially therapeutically targetable, angiogenesis-related molecules and, importantly, it has a distinctive angiogenic phenotype marked by a peculiar tumor-associated vasculature. Several studies, conducted in transgenic mouse models and in a large variety of human tumors of different histotype, clearly proved the substantial contribution of tumor-infiltrating myeloid cells, such as myeloid derived suppressor cells, monocytes and macrophages, in the formation and maintenance of abnormal blood vessels in tumors. By immunohistochemistry we thus explored the presence and the distribution of cells expressing myeloid markers in the inflammatory infiltrate of surgical treated metastatic ASPS. Indeed, we found that myeloid cells expressing CD14 and CD163 markers constitute the prominent cells in the inflammatory infiltrate of ASPS. These macrophage-like cells form a network surrounding the endothelial cells, or, interspersed in the tumor nest, they keep deep contact with tumor cells. In this commentary, we discussed our findings in relation to the recently published paper by Kummar and colleagues reporting the clinical and molecular results of a phase II clinical trial in patients with unresectable, metastatic ASPS treated with the anti-angiogenic drug cediranib, targeting the VEGFR-1,-2,-3 tyrosine kinases.

## Commentary

**Figure 1:**
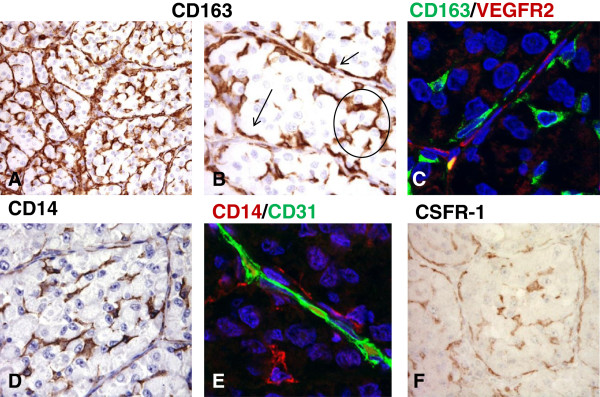
**Macrophages are key components of the inflammatory microenvironment in metastatic alveolar soft part sarcoma.** Immunohistochemical analysis of CD163+ cells infiltrating an untreated ASPS lesion **(A**-**B)**. As evidenced by the higher magnification image these cells are found in two distinct localizations: they are interspersed within nest tumor cells (circle) and they are also detectable in the perivascular region (arrows). **(B)** Confocal microscopy imaging of CD163+ cells (green) shows that they are aligned to endothelial VEGFR2+ cells (red) **(C)**. CD14 staining closely resembles that of CD163 **(D)** and double staining confirms that CD31+ endothelial cells (green) are lined by CD14+ macrophages (red). **(E)** A similar distribution of immunoreactivity is observed for CSFR-1 **(F)**.

We read with great interest the paper by Kummar et al. on cediranib in metastatic alveolar soft part sarcoma (ASPS) [1]. The study evaluated the antitumor activity of cediranib in 43 patients. The disease control rate (partial response plus stable disease) of patients who completed the therapy course was 84%. Tumor biopsies prior and after one week of cediranib were obtained from a subset of patients. Thus, the authors investigated for the first time the gene expression changes in ASPS after anti-angiogenic treatment, giving a comprehensive overview of the microarray/qRT-PCR profiles of significantly modulated genes in 7 validated cases. Included in this list are genes playing a direct role in the cancer driven neo-angiogenesis. Tumor lesions from patients treated with cediranib displayed a selective down-regulation of angiopoietin 2 (ANGPT2) and the up-regulation of its receptor TIE2. This opposite gene modulation may favor the angiopoietin 1 (ANGPT1)-TIE2 interaction and, together with the down-modulation of VEGFR1 (FLT1) and VEGFR2 (KDR), drive a physiological normalization of the vasculature at tumor site [2]. This hypothesis is also supported by the findings that genes encoding for proteins expressed by endothelial precursor cells or by the tumor-associated neo-vasculature such as Folate receptor 1 (FOLH1, known also as PMSA), CXCR7 and ESM1, [3-6] were also down-modulated. Gene profiling also revealed that in cediranib treated ASPS, CCL2 mRNA was associated to the presence of high level of CD163 gene expression. This coordinated gene up-regulation may suggest the selective recruitment of inflammatory monocytes that differentiate into fully mature macrophages at tumor site. However, CD163 is a marker associated to M2-like macrophages, endowed with pro-tumor and pro-angiogenic functions. Furthermore, the up-regulated expression of TIE2 in post treated samples can also be indicative of an increased accumulation of TIE2+ cells, known as vessel-associated macrophages, crucially involved in tumor-mediated neo-angiogenesis [7]. Thus, it is difficult to reconcile this complex scenario with the observation that the majority of the examined ASPS biopsies indeed derived from patients displaying a clinical response to treatment. The precise knowledge of the type, functional polarization and localization of immune tumor infiltrating cells in ASPS will possibly be of help in interpreting these data. While the Authors studied post-treatment changes on extractive tissues, we examined pre-treatment morphological and biochemical profiles of metastatic ASPS. We have investigated the pre-treatment immunophenotypic and biochemical profiles of 7 out of 15 patients treated with sunitinib since 2007 [8,9]. These 7 patients received surgery in our institute before treatment with sunitinib and their tumor tissues were available for immunohistochemistry (IHC) analysis. The other 8 patients underwent surgery elsewhere and material for the analyses was not at disposal. Moreover, ethical issues restrained the analysis of post-treated ASPS since there was no clinical indication for surgery after sunitinib. In a semi-quantitative scoring system, all the 7 samples displayed similar distribution and density for all the studied markers. An example, explicative for all the examined ASPS, is depicted in Figure 1. As showed in Figure 1, pre-treatment ASPS consisted of a sizeable population of CD163+ cells found in two distinct localizations. In fact, they were interspersed within nest tumor cells but, most importantly, they were also clearly detectable in the perivascular region where CD163+ cells (Figure 1 panels A and B, lower and higher magnification, respectively) were aligned to VEGFR2+ cells of endothelial nature (Figure 1, panel C: CD163 -NCL-CD163, Leica-Novocastra- green; VEGFR2 -55B11, Cell Signaling Technology- red). The CD163+ cells were also CD14+ (Figure 1, panel D) and therefore identifiable as tumor associated macrophages, and consequently aligned to CD31+ cells (panel E: CD14 -MS-1080-S1, Thermo Scientific- red; CD31 -JC70A, Dako- green). Of note, a similar distribution of immunoreactivity was observed for CSFR-1 (panel F: C20, sc-692, Santa Cruz Biotechnology). In addition, our previous investigation showed that CSFR-1 not only was expressed but also activated [8]. The CSF-1/CSF-1R signaling axis is the major regulator of survival, proliferation and functional differentiation of macrophages. All together, our observations established the presence of M2-like, CD163+ CD14+ macrophages in the tumor microenvironment of naive ASPS. These myeloid cells are active inflammatory components that may promote VEGF-mediated vasculogenesis and, although not physically part of the vasculature, they are thought to provide trophic support to the characteristic ASPS vascular network. The pre-treatment immunophenotypic ASPS signature we observed strongly suggest that myeloid immune component of the ASPS microenvironment may directly influence the response to anti-angiogenic therapies and become direct target for anti-VEGF/VEGFR drugs, such as cediranib. However, Kummar’s data indicated that the CCL2 and CD163 genes, known markers of inflammatory myeloid cell infiltration and associated with M2-like pro-tumor macrophages, were boosted in those ASPS of patients treated with cediranib with evidence of response. The absence of drug-induced down-regulation in the myeloid inflammatory components raises questions concerning its possible association with the profile of the tumor, with response or resistance to treatment. At first, we can argue that cediranib treatment may induce a functional shift of the infiltrating myeloid cells instead of modulating their frequency at tumor site (that would have ended up with a diminished expression of genes encoding for markers of myeloid cells). Indeed, the active role of VEGF/VEGFR signaling in the functional generation of myeloid cells with strong pro-angiogenic and immunosuppressive functions is amply documented both in animal models and in humans [10]. Thus, by blocking this pathway, cediranib might affect the type or the functional status of the inflammatory cells, eventually contributing to the transformation of the immunosuppressive, pro-angiogenic microenvironment into a more immunostimulatory, anti-tumor milieu [11]. In support of this interpretation, cediranib up-regulated the inflammatory pathway genes controlled by the nuclear factor-kB, as highlighted by the authors themselves. This strong and coordinated boost of inflammation-related genes might transform the chronic, pro-tumorigenic inflammation at the tumor site into an acute inflammation status that is perceived by the immune system as ‘dangerous’ and is generally correlated with an active, protective immune response [12]. In addition, as recently reported in different tumor settings [13], anti-angiogenic therapy induces a vascular normalization that alleviates tumor local hypoxia, thus removing one of the major factors responsible for the generation of an immunosuppressive environment [14]. As an alternative hypothesis, it cannot be excluded that M2-like pro-angiogenic myeloid cells present at tumor site, as shown by our immunophenotyping studies, might increase in number to counteract the massive, antiangiogenic-mediated vascular pruning. Indeed, in such a case, M2 polarized myeloid cells could be the immune-related mediators of acquired resistance. To decipher the role of this ‘inflammatory’ component in ASPS treated with cediranib, and, more in general, for the anti-angiogenic therapies of solid tumors, it will be crucial to assess whether or not the localization and the functional activation of the myeloid cells, resident or newly recruited, in treated ASPS overlap with those found in pre-treated tumors. This matter can only be dissected through a thorough pre/post-treatment analysis of pair-matched ASPS samples. Nonetheless, the gene expression changes induced in ASPS after cediranib treatment and the presence of a structured myeloid cell infiltration provide the rationale for further studies to investigate the feasibility of approaches targeting myeloid cells in combination with anti-angiogenic therapy. Drugs limiting the viability, function and differentiation of cells of myeloid lineage have been recently introduced in clinical setting. Among them all-trans-retinoic acid (ATRA), with differentiation potential, and synthetic triterpenoids, that reduce the intracellular reactive oxygen species, molecules mediating the suppressive function of MDSC and macrophages, have been recently used in pilot clinical studies [15,16]. Furthermore, trabectedin has been recently shown to limit the viability of monocytes and tumor-associated macrophages in sarcomas [17], and bisphosphonates, employed in the treatment of bone metastasis, may also potentially target macrophages [18]. In conclusion, the precise knowledge of the nature of tumor infiltrating cells before and after a given drug treatment may pave the way to new combined therapies aimed at overcoming drug induced resistance.

## Competing interests

SS has received research funding by Pfizer for clinical study. All other authors declare they have no competing interests.

## Authors’ contributions

CC, MT, TN, BV, LR, SS and SP made intellectual contributions and drafted the manuscript. BV designed and performed immunohistochemical and confocal analysis. All authors read and approved the final manuscript.
